# Larval habitats of the *Anopheles farauti* and *Anopheles lungae* complexes in the Solomon Islands

**DOI:** 10.1186/s12936-016-1196-7

**Published:** 2016-03-15

**Authors:** Tanya L. Russell, Thomas R. Burkot, Hugo Bugoro, Allan Apairamo, Nigel W. Beebe, Weng K. Chow, Robert D. Cooper, Frank H. Collins, Neil F. Lobo

**Affiliations:** Australian Institute of Tropical Health and Medicine, James Cook University, Cairns, QLD 4870 Australia; National Vector Borne Disease Control Programme, Ministry of Health, Honiara, Solomon Islands; School of Biological Sciences, University of Queensland, St. Lucia, QLD 4068 Australia; CSIRO, Dutton Park, Brisbane, QLD 4102 Australia; Australian Army Malaria Institute, Gallipoli Barracks, Enoggera, 4052 Australia; Eck Institute for Global Health, Department of Biological Sciences, University of Notre Dame, Notre Dame, IN 46556 USA

**Keywords:** Solomon Islands, Malaria, *Anopheles farauti*, Species distribution, Density dependent development

## Abstract

**Background:**

There is an urgent need for vector control tools to supplement long-lasting insecticidal nets (LLINs) and indoor residual spraying; particularly in the Solomon Islands where the primary vector, *Anopheles farauti*, is highly anthropophagic and feeds mainly outdoors and early in the evening. Currently, the only supplementary tool recommended by the World Health Organization is larval source management (LSM). The feasibility and potential effectiveness of LSM requires information on the distribution of anophelines, the productivity of larval habitats and the potential impacts of larval control on adult fitness.

**Methods:**

The distribution of anophelines in Central and Western Provinces in the Solomon Islands was mapped from cross-sectional larval habitat surveys. The composition and micro-distribution of larval instars within a large permanent river-mouth lagoon was examined with a longitudinal survey. Density-dependent regulation of *An. farauti* larvae was investigated by longitudinally following the development and survival of different densities of first instars in floating cages in a river-mouth lagoon.

**Results:**

Five anopheline species were molecularly identified from a range of fresh and brackish water habitats: *An. farauti s.s.*, *An. hinesorum*, *An. lungae*, *An. nataliae* and *An. solomonis*. The most common habitats used by the primary malaria vector, *An. farauti*, were coastal lagoons and swamps. In the detailed study of lagoon micro-productivity, *An. farauti* was non-uniformly distributed with highest densities found at collections sites most proximal and distal to the mouth of the lagoon. The survival of *An. farauti* larvae was more than twofold lower when larvae were held at the highest experimental density (1 larva per 3.8 cm^2^) when compared with the lowest density (1 larva per 38 cm^2^).

**Conclusions:**

The only documented major malaria vector collected in larval surveys in both Central and Western Provinces was *An. farauti*. Lagoons and swamps, the most common, largest and (potentially) most productive larval sites of this malaria vector, were “few, fixed and findable” and theoretically, therefore, amenable to successful LSM. However, the immense scale and complexity of these ecosystems in which *An. farauti* larvae are found raises questions regarding the ability to effectively control the larvae, as incomplete larviciding could trigger density dependent effects resulting in increased larval survivorship. While LSM has the potential to significantly contribute to malaria control of this early and outdoor biting vector, more information on the distribution of larvae within these extensive habitats is required to maximize the effectiveness of LSM.

## Background

The Solomon Islands is currently implementing country-wide intensified malaria control using universal distribution of long-lasting insecticidal nets (LLINs) and indoor residual spraying (IRS). Unfortunately, the main malaria vector in the Solomon Islands, *Anopheles farauti*, displays behavioural resistance to indoor vector control by blood feeding predominantly when people are outdoors [1]. This behavioural shift first occurred in response to IRS with DDT in the 1970s [2, 3] and has persisted in the Solomon Islands with LLINs being the primary malaria vector control strategy [4–7]. Despite the early and outdoor biting behaviour of *An. farauti*, LLINs and IRS have had a significant impact on malaria transmission. However, achieving malaria elimination will require additional vector control to minimize outdoor transmission. The only outdoor strategy recommended by the World Health Organization is larval source management (LSM) [8] and this has the potential to limit transmission both indoors and outdoors. Larval source management is only recommended in areas where the larval habitats are few in number, fixed in location and easily accessible [9]. To ascertain the feasibility of implementing LSM in the Solomon Islands, information on the types of larval habitats utilized by vectors including their location in proximity to villages is needed.

Nine species of anophelines occur in the Solomon Islands: six members of the *An. punctulatus* group: *An. farauti*, *An. irrenicus, An. hinesorum,An. punctulatus*, *An. koliensis* and *An. rennellensis* [10, 11]; as well as three members of the *An.lungae* complex: *An. lungae*, *An. solomonis* and *An. nataliae* [12]. Of these, the only known malaria vectors in the Solomon Islands are *An. farauti*, *An.punctulatus* and *An. koliensis*. *Anophelespunctulatus* and *An*. *koliensis* became uncommon after IRS with DDT was extensively used for malaria vector control tool in the 1970s [13]. Larvae of *An. farauti*, are found within a kilometre of the coast in both fresh and brackish water (≥70 % seawater) [14, 15]. Freshwater larval habitats of *An. farauti* include both natural and man-made depressions such as drains, vehicle tracks, foot prints, pig wallows and ground-pools [5, 16] that are dependent on rainfall [17, 18]. Large numbers of *An. farauti* are believed to be associated with large, permanent, brackish water lagoons or swamps that form behind sandbars that block the flow of water into the sea [17–19] as high adult biting densities and malaria parasite rates are associated with villages proximal to these coastal habitats [20, 21].

The population dynamics of mosquitoes are influenced by both intrinsic and exogenous processes [22–25]. If density effects operate on mosquito larvae in large larval habitats, the impact of interventions targeting anopheline larval abundance will be disproportionate to the density of the anopheline populations’ (linear reductions in populations may not result in linear reductions in productivity or fitness). The majority of studies on density-dependent regulation of mosquito larvae in small aquatic habitats were conducted under controlled laboratory or in “semi-field” conditions. Generally, these studies have shown that phenotypic traits which mediate individual fitness (e.g. body size) in larvae and adults are optimized at low larval population densities [26–28]. Studies to define density-dependence of mosquitoes in large larval habitats are needed. In the Solomon Islands, *An. farauti* use large lagoons as well as smaller and more temporary aquatic sites as larval habitats [19]. Prior to attempting larval control in the Solomon Islands, data on the distribution and abundance of categories of larval habitats are required and the potential role of larval density dependence on adult fitness needs to be defined.

## Methods

### Study sites

The study was conducted in Central and Western Provinces in the Solomon Islands where small villages are mainly found in coastal areas. The climate of the region is hot and wet; the median annual rainfall in Central Province is 2837 mm (based on 43 years of data collected from Tulagi; [29]). Annual rainfall estimates for Western Province are 2667 mm from Gizo (based on 28 years of data up to 1952; [29]) and 3725 mm from Munda (based on 11 years of data from 1999–2009; Solomon Islands Bureau of Meteorology, Unpublished data). Although rain falls throughout the year, relatively less rain falls between May to September. The mean annual temperature on the coast is 26 °C and is reasonably constant throughout the year. Mean daily temperatures range between 24 and 30 °C. Malaria is primarily transmitted by *An. farauti s.s*.

### Larval distribution

The distribution of anophelines was investigated by larval presence-absence surveys. The surveys were focused within 2 km of the coast line, as this is where villages are located. Larval habitats were categorized as one of six classes and locations recorded by GPS. Surveys were conducted from August to September 2011 in Central Province (9°0′S, 159°45′E) and in February and May 2013 in Western Province (8°0′S, 157°0′E). In Central Province, 14 villages were surveyed on the islands of Ngella Sule and Tulagi Islands. In Western Province, 54 villages were surveyed on the islands of Vella Lavella, Ranonnga, Gizo, Kolombangara, Vonavona, Kohinggo and New Georgia Islands. Potential larval habitats were sampled with 250 ml dippers and larval samples were stored in 70 % ethanol in micro-centrifuge tubes for subsequent identification by molecular analysis of the internal transcribed spacer region II (ITS2) of the ribosomal DNA [30].

### Lagoon micro-habitats

**Fig. 1:**
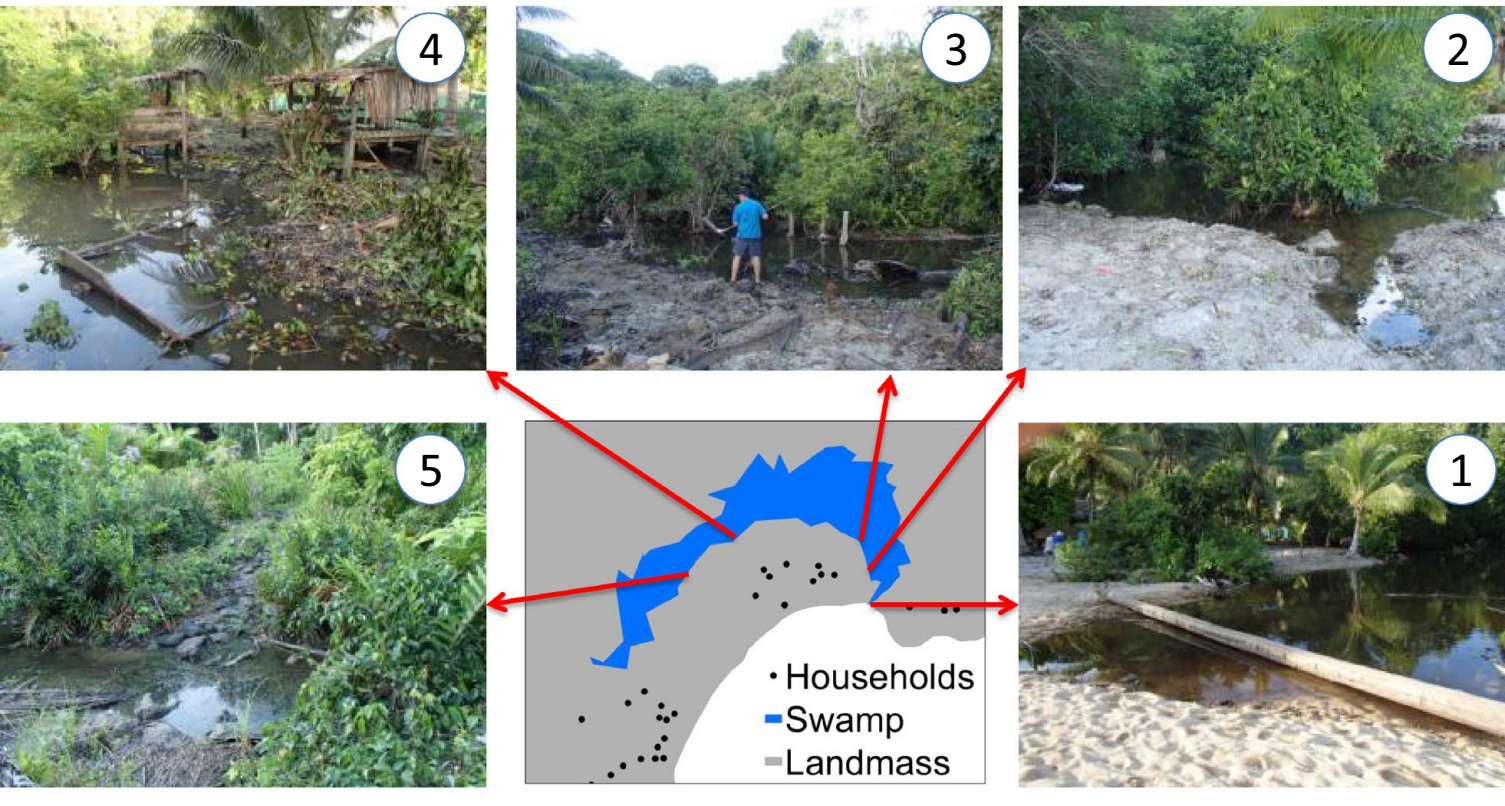
Images and locations of the sampling stations used to examine micro-productivity of *An. farauti* larvae in Haleta Village, Central Province, Solomon Islands

The micro-distribution of larval instars at different sites within a large permanent river-mouth lagoon in Haleta village, Central Province, Solomon Islands was evaluated [7]. The lagoon was a known larval habitat that forms when surface water run-off accumulates behind a sand bar that prevents drainage to the sea. After periods of heavy rain, the sandbar breaks and releases the accumulated water to the ocean and the area of the larval habitat is reduced. Hereafter, this larval habitat will be referred to as the lagoon. The numbers of the different anopheline larval instars were monitored daily at five stations (1 × 5 m; Fig. 1) along the northern edge of the lagoon over 10 consecutive days from the 2nd–11th December 2012. Each station was sampled once daily by 10 dips with a standard 250 ml dipper. The number of larvae by instar was recorded per dip. The water temperature and salinity was concurrently measured at each station with a thermometer and a hand held refractometer (Atago Co. Ltd, Japan), respectively.

### Density dependent development

**Fig. 2:**
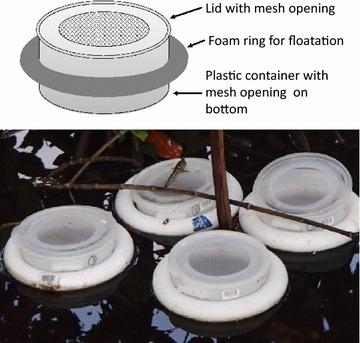
Replicate mesh cages used to manually manipulate the densities of 1st instar *An. farauti* within the lagoon in Haleta Village, Central Province, Solomon Islands

Density-dependent regulation of mosquito larvae was investigated by seeding first instar *An. farauti* larval at different densities into floating larval cages encompassing a surface are of 380 cm^2^ (diameter 22 cm; Fig. 2). The larval cages permitted exchange of water and microfora with the habitat in which they were placed but excluded the entry of predators or other fauna. The larval cages tracked the temperature, nutrition and other environmental conditions in the permanent larval habitat in which they were placed.

Wild-caught female *An. farauti* captured by human landing catches (HLC) (see [1, 7, 31]) were used to generate the F1 first instars used here. Ethical approvals for conducting HLC were obtained from the National Health Research and Ethics Committee, Solomon Islands (02-05-2011), the James Cook University Human Research Ethics Committee, Australia (H4122) and the University Hospitals Case Medical Centre Institutional Review Board for Human Investigation, USA (05-11-11). Blood-fed specimens from HLCs were isolated and placed into 70 ml plastic specimen jars holding a piece of damp cotton-wool covered with filter paper on the bottom as an oviposition substrate. The top of each oviposition chamber was covered with mosquito netting overlaid with damp cotton-wool to ensure high humidity. Filter papers with eggs wer transferred into a petri dish containing rain water for hatching. F1 first instars from wild-caught females were mixed and allocated to larval cages at densities of 10, 25, 50 and 100 per larval cage (i.e. 1 larva per 38, 15.2, 7.6 and 3.8 cm^2^, respectively) with five replicates of each density. For each treatment group the survival of larvae was monitored daily over 10 consecutive days from the 2nd–11th December 2012. Larval survival was defined as total larval present at a time point divided by the total released into the cage.

### Statistical analysis

For the larval distribution study, three related data sources were created: (1) the geo position of each site surveyed (shapefile); (2) the field survey conducted during each site survey (tabular); and (3) the molecular data containing the species identifications of the larval samples (tabular) [32]. The data for each source was linked with a unique identifier that was allocated to each site during the PDA-based survey.

For lagoon micro-productivity, a dataset was constructed that detailed the total number of anophelines per dip, and scored for presence as 0 (negative) or 1 (positive for larvae) for each dip [32]. The influence of location (sampling stations) on both the presence and density of larvae was analysed with generalized linear models (GLMs). The influence of the two environmental factors, water temperature and salinity, with both the presence and density of larvae was analysed with generalized linear mixed models (GLMMs) with location as a subject variable to account for repeated sampling. The distributions for the models were: (1) binary data (presence or absence) fitted to a binomial distribution with a logit link function and (2) count data (density) fitted to a negative binomial distribution with a log link function because data were not normally distributed.

The density-dependent regulation of mosquito larvae was analysed using a Cox regression to compare the survival of mosquito larvae held at different densities [32]. The Cox regression determined the relative risk of dying (hazard ratios) for each density group compared with the lowest density tested (10 larvae per cage [1 larva per 38 cm^2^]). The model was weighted by the replicate number to account for longitudinal sampling. All analyses were conducted using the *R* package V3.1.2 [33].

## Results

### Larval distribution

**Fig. 3:**
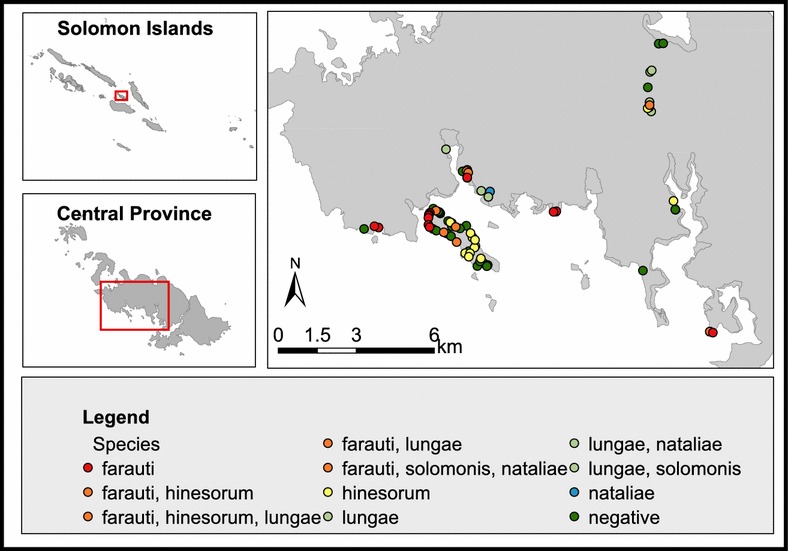
Species distribution of Anopheline fauna based on larval surveys conducted in Central Province, Solomon Islands. The Islands included in the survey were Ngella Sule and Tulagi Islands

**Fig. 4:**
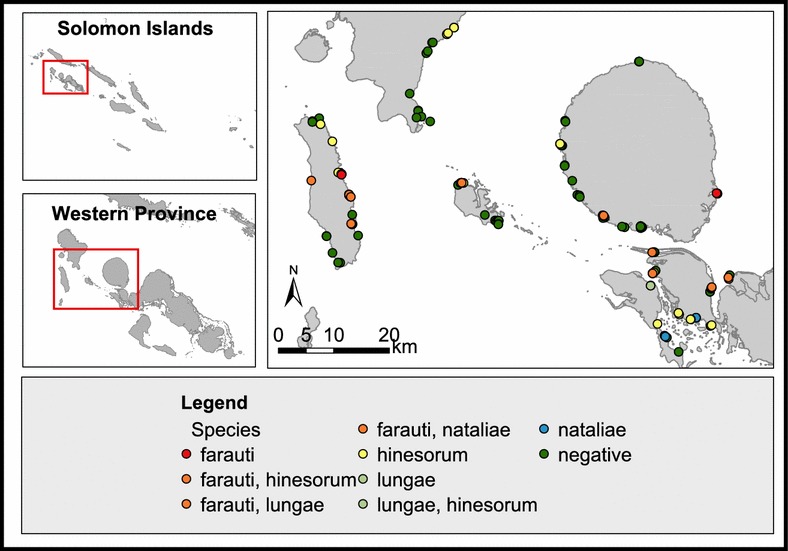
Species distribution of Anopheline fauna based on larval surveys conducted in Western Province, Solomon Islands. The Islands included in the survey were Vella Lavella, Ranonnga, Gizo, Kolombangara, Vonavona, Kohinggo and New Georgia Islands

Anopheline larvae were collected from 108 larval habitats (58 sites in Central Province and 50 sites in Western Province) (Figs. 3, 4). Overall 391 specimens of five species were identified by PCR: *An. farauti s.s.*, *An. hinesorum, An. lungae, An. nataliae* and *An. solomonis*. *Anopheles farauti s.s.* and *An. hinesorum* were the most abundant and widespread species found in both provinces. *Anopheles lungae* and *An. nataliae* were present but less common in both provinces. *Anopheles solomonis* was only found in Central Province. The anophelines were found across a range of larval habitats: coastal lagoons and swamps, drains, transient pools, man-hade holes, riverine and spring wells (Table 1). Both *An. farauti s.s.* and *An. hinesorum* were found in all habitat classes, with the highest prevalence habitat being coastal lagoons and swamps. The most commonly used habitat for *An. lungae* was riverine areas.

**Table 1:** Aquatic larval habitats utilized by the five anopheline species found in Central and Western Provinces, Solomon Islands

Habitat type	Species and number of sites occupied (%)				
	*An. farauti*	*An. hinesorum*	*An. lungae*	*An. nataliae*	*An. solomonis*
Lagoon or swamp	19 (55.9)	20 (45.5)	4 (26.7)	4 (40.0)	0 (0.0)
Drains	3 (8.8)	8 (18.2)	1 (6.7)	0 (0.0)	0 (0.0)
Transient pools	2 (5.9)	1 (2.3)	3 (20.0)	0 (0.0)	0 (0.0)
Man-made holes	7 (20.6)	5 (11.4)	0 (0.0)	1 (10.0)	1 (50.0)
Riverine	1 (2.9)	7 (15.9)	7 (46.7)	4 (40.0)	1 (50.0)
Spring well	2 (5.9)	3 (6.8)	0 (0.0)	1 (10.0)	0 (0.0)

### Lagoon micro-productivity

A total of 408 anopheline larvae were collected; 56 % (n = 227) were early instars (I–II) and 44 % (n = 181) were late instars (III–IV). The location of the sampling station along the length of the lagoon influenced both the presence (χ^2^ = 13.26, df = 4, p = 0.001) and density (χ^2^ = 22.92, df = 4, p < 0.001) of larvae per dip. The larval density was highest at the monitoring stations most proximal and distal from the sandbar behind which the lagoon formed (stations 1 and 5; Table 2). The water temperature was fairly uniform across the sampling stations, ranging from 30.8 to 31.6 °C, with salinity diminishing from 1 ppt at the station closet to the sandbar to 0 ppt at the site most distal from the sandbar (Table 2). Evidence for an impact of either presence or density of larvae by either water temperature (β = 0.37, df = 43, p = 0.359; β = 0.001, df = 43, p = 0.995, respectively) or salinity (β = −0.11, df = 43, p = 0.904; β = 0.109, df = 43, p = 0.301, respectively) was not found.

**Table 2:** Spatial comparison of *An. farauti* larval occurrence and environmental factors recorded in the study lagoon in Haleta Village, Central Province, Solomon Islands

	Larval occurrence		Environmental factors	
Location	Density per dip^a^	Presence (daily)^b^	Temp (°C)	Salinity (ppt)
1	1.79 ± 0.39	1.0 ± 0.0	31.6 ± 0.3	1.0 ± 0.7
2	0.73 ± 0.15	0.8 ± 0.1	31.0 ± 0.3	0.6 ± 0.6
3	0.26 ± 0.06	0.8 ± 0.1	30.9 ± 0.2	0.4 ± 0.4
4	0.20 ± 0.07	0.5 ± 0.2	31.1 ± 0.3	0.0 ± 0.0
5	1.10 ± 0.24	1.0 ± 0.0	30.8 ± 0.3	0.0 ± 0.0

Data presented are mean ± se

^a^ Mean larval density per dip over 10 days

^b^ Presence of mosquito larvae was defined as the proportion of 10 dips in which larvae of any stage were present

### Density dependent development in the lagoon

**Fig. 5:**
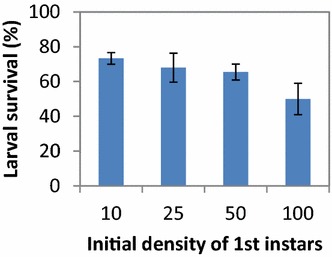
The survival of *An. farauti* larvae after being held in mesh cages for 9 days at different densities. Note: larval survival was defined as total larval present divided by the total released into the cage

The survival of *An. farauti* larvae was more than twofold lower when larvae were held at the highest experimental density (100 per cage or 1 larva per 3.8 cm^2^) when compared with the lowest density (10 per cage or 1 larva per 38 cm^2^; Hazard ratio [HR] = 2.11, se = 0.21, *p* = 0.0003; Fig. 5). The survival of larvae at densities of 25 (1 larva per 15.2 cm^2^) and 50 (1 larva per 7.6 cm^2^) per cage was not significantly different from 10 per cage (25 larvae: HR = 1.24, se = 0.23, *p* = 0.351; 50 larvae: HR = 1.45, se = 0.21, *p* = 0.082).

For the lowest experimental density (10 per cage) the surviving larvae (n = 31) all pupated with a cumulative 14 adults emerging by day 9 following larval release into the cages. The development rate was delayed at higher densities. Adults emergence was not seen from any of the other densities with the exception of two undersized (by visual inspection) adults from the highest density cage (holding 100 larvae). Only 8 of the surviving 68 (11.7 %) larvae pupated by day 10 in the 25 larvae per cage experimental density while only 10 of the surviving 131 larvae (7.6 %) pupated in the 100/cage experimental density. Larval and pupal sizes diminished with increasing density based on a visual inspection.

## Discussion

During mosquito surveys conducted in the early 1970s, *An. farauti*, *An. punctulatus* and *An. koliensis* were found on all the main islands in the Solomon Islands except Temotu Province [13, 34]. In the Solomon Islands, extensive DDT-IRS was conducted during the 1960s and 1970s and had a significant impact on population densities: after repeated spray rounds these highly endophagic species became difficult to find [2, 34, 35]. Both *An. punctulatus* and *An. koliensis* were found on Malaita in 1987 [36], with this being the last record of *An. koliensis* in the Solomon Islands. *Anopheles punctulatus* was found during the 1990s on both Guadalcanal and Malaita [10, 37]. Mosquito surveys have not been conducted since the early 1970s in Central Province [3]. In Western Province, a limited survey was conducted (in Titiana village) during the early 1990s and only *An. farauti* was found [37]. The malaria vectors, *An. punctulatus* and *An. koliensis*, were not identified in this study during the extensive larval habitat surveys in Central and Western Provinces.

After the 2009 faunal surveys in Santa Isabel, it was proposed that increased competition for larval sites by *An. hinesorum* may have inhibited the prevalence and range of *An. punctulatus* and *An. koliensis* [5]. This is significant for malaria transmission because *An. hinesorum* in the Solomon Islands is primarily zoophagic (e.g., a non-vector of human malaria) [5, 10, 37]. On Santa Isabel, Central and Western Provinces, *An. hinesorium* occupied sites normally associated with *An. punctulatus* and *An. koliensis* such as drains and semi-permanent ground pools. The larval surveys in this study only focused on coastal areas because this is where most villages (and malaria) are found and this would have excluded freshwater sites distant from the coast.

The distribution of *An. farauti* larvae was not uniform among five sampling sites within a large coastal lagoon. The density and presence of larvae was highest at the proximal and distal sites relative to the sandbar that created the lagoon but this was not associated with either temperature or salinity. Similar studies on Guadalcanal during 2007–08 [19] also found that *An. farauti* distribution was not uniform within large coastal larval habitats. While the habitats in Guadalcanal and Central Provinces were both coastal and were formed when water flow into the ocean was blocked by a sand mouth, the Guadalcanal sites were larger streams that were blocked by sandbars whereas the site in Central Province was a lagoon resulting from surface water runoff trapped by a sandbar from flowing into the ocean. On Guadalcanal the density and prevalence of larvae was highest near the mouth of the steam and declined as the sampling stations moved inland where the water became deeper and faster flowing. On Guadalcanal, larval densities were also positively associated with aquatic emergent plants and filamentous algae [19].

Evidence for potential density dependent development effects were seen within the river-mouth lagoon. The survival of larvae at the highest density in the cages was nearly two-fold less than that when held at the lowest density. Confirming density dependent impacts will require careful quantitative documentation of the density of larvae in natural habitats and the impacts of density on adult mosquito fitness; in addition potential density dependent impacts will need to be teased apart from exogenous influences. The implications of density dependent effects for malaria control remain unclear. Potentially, if anopheline growth is under strong density dependent regulation, control measures may become proportionately less effective as larval densities diminish because the remaining individuals could compensate with enhanced reproduction and survival [38, 39]. Understanding the concurrent roles of exogenous and density dependent factors on population growth is crucial for predicting the response of vector populations to control strategies.

## Conclusion

Anopheline surveys in two provinces found an extensive distribution of *An. farauti* but did not find either *An. punctulatus* or *An. koliensis.* This suggests that these two formerly dominant malaria vectors, *An. punctulatus* and *An. koliensis*, are uncommon if not eliminated from Central and Western Provinces. The primary vector, *An. farauti*, remains and has a habit of feeding early and outdoors when humans are not protected by LLINs and IRS. While the primary larval habitat of *An. farauti* in the Solomon Islands are river-mouth lagoons and large swamps which are “few (in number), fixed (permanent) and findable (located close to villages)” [40] and thereby fulfil, in theory, the attributes that should make these larval habitats amenable to LSM, it is unclear if the large size of these habitats are “fixable” without more information on the distributions and densities of larvae within the complex habitats (swamps encompassing extensive vegetation an multiple microhabitats) that they occupy.

## Availability of data and materials

The datasets supporting the conclusions of this article are available in the James Cook University Tropical Data Hub repository: 10.4225/28/56C65.

## Abbreviations

GLM: generalized linear model
GLMM: generalized linear mixed model
HLC: human landing catch
HR: hazard ratio
IRS: indoor residual spraying
LLINs: long-lasting insecticidal nets
LSM: larval source management
